# 1-(6-Fluoro-1,3-benzothia­zol-2-yl)-3-phenyl-1*H*-pyrazole-4-carbaldehyde

**DOI:** 10.1107/S1600536811049634

**Published:** 2011-11-30

**Authors:** Hoong-Kun Fun, Chin Wei Ooi, D. Munirajasekhar, M. Himaja, B. K. Sarojini

**Affiliations:** a X-ray Crystallography Unit, School of Physics, Universiti Sains Malaysia, 11800 USM, Penang, Malaysia; b Pharmaceutical Chemistry Division, School of Advanced Sciences, VIT University, Vellore 632 014, Tamil Nadu, India; c Department of Chemistry, P. A. College of Engineering, Nadupadavu 574 153 D. K., Mangalore, India

## Abstract

The asymmetric unit of the title compound, C_17_H_10_FN_3_OS, consists of two crystallographically independent mol­ecules. In one mol­ecule, the pyrazole ring makes dihedral angles of 6.51 (7) and 34.02 (9)°, respectively, with the terminal 1,3-benzothia­zole ring system and the phenyl ring, while in the other mol­ecule these values are 6.41 (8) and 23.06 (9)°. In the crystal, the molecules are linked by weak π–π [centroid–centroid distance = 3.7069 (10) Å] and C—H⋯π inter­actions.

## Related literature

For the biological activity of benzothia­zole derivatives, see: Al-Soud *et al.* (2006 ▶); Kini *et al.* (2007 ▶); Munirajasekhar *et al.* (2011 ▶); Gurupadayya *et al.* (2008 ▶); Bowyer *et al.* (2007 ▶); Mittal *et al.* (2007 ▶); Rocío Pozas *et al.* (2005 ▶); Rana *et al.* (2008 ▶). For a related structure, see: Fun *et al.* (2011 ▶). For bond-length data, see: Allen *et al.* (1987 ▶).
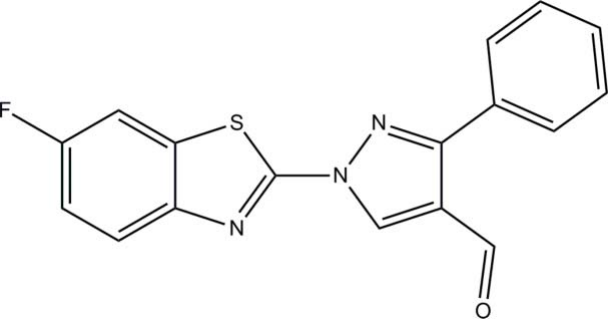


## Experimental

### 

#### Crystal data

C_17_H_10_FN_3_OS
*M* *_r_* = 323.34
Triclinic, 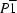

*a* = 8.0994 (3) Å
*b* = 13.6566 (4) Å
*c* = 13.8472 (5) Å
α = 70.393 (1)°
β = 85.264 (1)°
γ = 89.069 (1)°
*V* = 1437.80 (9) Å^3^
*Z* = 4
Mo *K*α radiation
μ = 0.24 mm^−1^
*T* = 296 K
0.50 × 0.42 × 0.23 mm

#### Data collection

Bruker APEX DUO CCD area-detector diffractometer
Absorption correction: multi-scan (*SADABS*; Bruker, 2009 ▶) *T*_min_ = 0.887, *T*_max_ = 0.945
31117 measured reflections
8251 independent reflections
6347 reflections with *I* > 2σ(*I*)
*R*_int_ = 0.024

#### Refinement

*R*[*F* ^2^ > 2σ(*F* ^2^)] = 0.043
*wR*(*F* ^2^) = 0.144
*S* = 1.07
8251 reflections
415 parameters
H-atom parameters constrained
Δρ_max_ = 0.31 e Å^−3^
Δρ_min_ = −0.24 e Å^−3^

### 

Data collection: *APEX2* (Bruker, 2009 ▶); cell refinement: *SAINT* (Bruker, 2009 ▶); data reduction: *SAINT*; program(s) used to solve structure: *SHELXTL* (Sheldrick, 2008 ▶); program(s) used to refine structure: *SHELXTL*; molecular graphics: *SHELXTL*; software used to prepare material for publication: *SHELXTL* and *PLATON* (Spek, 2009 ▶).

## Supplementary Material

Crystal structure: contains datablock(s) global, I. DOI: 10.1107/S1600536811049634/is5013sup1.cif

Structure factors: contains datablock(s) I. DOI: 10.1107/S1600536811049634/is5013Isup2.hkl

Supplementary material file. DOI: 10.1107/S1600536811049634/is5013Isup3.cml

Additional supplementary materials:  crystallographic information; 3D view; checkCIF report

## Figures and Tables

**Table 1:** Hydrogen-bond geometry (Å, °) *Cg*4 is the centroid of the C11*B*–C16*B* ring.

*D*—H⋯*A*	*D*—H	H⋯*A*	*D*⋯*A*	*D*—H⋯*A*
C5*B*—H5*BA*⋯*Cg*4^i^	0.93	2.85	3.4757 (19)	126

Symmetry code: (i) 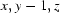.

**Fig. 1.**
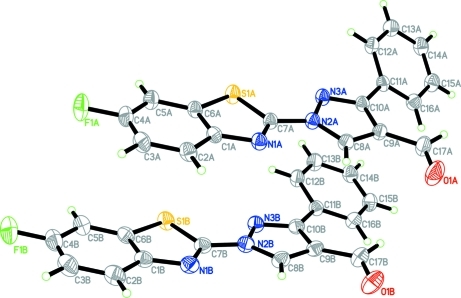
The molecular structure of the title compound, showing two independent molecules with atom labels and 30% probability displacement ellipsoids.

**Fig. 2.**
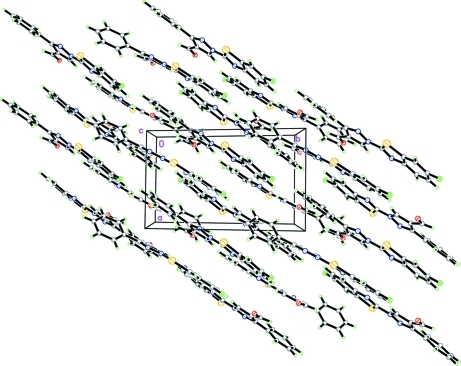
The crystal packing of the title compound.

## supplementary crystallographic information

### Comment

Benzothiazoles are very important bicyclic ring compounds which are of great interest because of their biological activities. The substituted benzothiazole derivatives have emerged as significant components in various diversified therapeutic applications. The literature review reveals that benzothiazoles and their derivatives show considerable activity including potent inhibition of human immunodeficiency virus type 1 (HIV-1) replication by HIV-1 protease inhibition (Al-Soud *et al.*, 2006), antitumor (Kini *et al.*, 2007), anthelmintic (Munirajasekhar *et al.*, 2011) analgesic and anti-inflammatory (Gurupadayya *et al.*, 2008), antimalarial (Bowyer *et al.*, 2007), antifungal (Mittal *et al.*, 2007), anticandidous (Rocío Pozas *et al.*, 2005) as well as various *CNS* activities (Rana *et al.*, 2008). The structure of 2-[5-(4-methoxyphenyl)-3-phenyl-4,5- dihydro-*1H*-pyrazol-1-yl]-6-methyl-1,3-benzothiazole has been reported by Fun *et al.* (2011). The present work describes the synthesis and crystal structure of the title compound, 1-(6-fluorobenzo-1,3-thiazol-2-yl)-3-phenyl-*1H*-pyrazole-4- carbaldehyde which was prepared from the reaction of 1-(6-fluoro-1,3-benzothiazol-2-yl)-2-(1-phenylethylidene)hydrazine treated with Vilsmeier-Haack reagent to obtain crystals of the title compound.

The asymmetric unit of the title compound consists of two crystallographically independent molecules A and B as shown in Fig. 1. The pyrazole rings (N2A/N3A/C8A–C10A and N2B/N3B/C8B–C10B) are approximately planar with a maximum deviation of 0.002 (2) Å for atom C8A and 0.001 (2) Å for atom C9B. The central pyrazole (N2/N3/C8–C10) ring makes dihedral angles of 6.51 (7) and 34.02 (9)°, respectively, with the terminal benzo[*d*]thiazole ring (S1/N1/C1–C7) system and the phenyl ring (C11–C16) for molecule A. These values are 6.41 (8) and 23.06 (9)° for molecule B. The bond lengths (Allen *et al.*, 1987) and angles are within normal ranges and are comparable to the related structure (Fun *et al.*, 2011).

In the crystal structure (Fig. 2), no classical hydrogen bonds were observed and stabilization is provided by a weak π–π interaction between the thiazoline (S1A/N1A/C1A/C6A–C7A) and the phenyl ring (C11B–C16B) [centroid-to-centroid (-1 + *x*, *y*, *z*) distance = 3.7069 (10) Å]. The structure is further stabilized by C—H···π interactions, involving the centroid of phenyl ring (C11B–C16B; *Cg*4; Table 1).

### Experimental

1-(6-Fluorobenzo[*d*]thiazol-2-yl)-2-(1-phenylethylidene)hydrazine was treated with Vilsmeier-Haack reagent (DMF/POCl_3_: 10:1.1 ml) and was stirred at 60–65 °C for 2.5 h. It was poured into cold water and the solid that separated out by neutralization with NaHCO_3_ was filtered, washed with water and was then purified by column chromatography. The product was recrystallized from petroleum ether and ethyl acetate (80: 20) to yield block-shaped colorless crystals.

### Refinement

All the H atoms were positioned geometrically (C—H = 0.93 Å) and refined using a riding model, with *U*_iso_(H) = 1.2*U*_eq_(C).

### Crystal data

**Table:**

C_17_H_10_FN_3_OS	*Z* = 4
*M**_r_* = 323.34	*F*(000) = 664
Triclinic, *P*1	*D*_x_ = 1.494 Mg m^−^^3^
Hall symbol: -P 1	Mo *K*α radiation, λ = 0.71073 Å
*a* = 8.0994 (3) Å	Cell parameters from 9988 reflections
*b* = 13.6566 (4) Å	θ = 2.6–32.6°
*c* = 13.8472 (5) Å	µ = 0.24 mm^−^^1^
α = 70.393 (1)°	*T* = 296 K
β = 85.264 (1)°	Block, colourless
γ = 89.069 (1)°	0.50 × 0.42 × 0.23 mm
*V* = 1437.80 (9) Å^3^	

### Data collection

**Table:**

Bruker APEX DUO CCD area-detector diffractometer	8251 independent reflections
Radiation source: fine-focus sealed tube	6347 reflections with *I* > 2σ(*I*)
graphite	*R*_int_ = 0.024
φ and ω scans	θ_max_ = 30.0°, θ_min_ = 1.6°
Absorption correction: multi-scan (*SADABS*; Bruker, 2009)	*h* = −11→11
*T*_min_ = 0.887, *T*_max_ = 0.945	*k* = −18→19
31117 measured reflections	*l* = −19→19

### Refinement

**Table:**

Refinement on *F*^2^	Primary atom site location: structure-invariant direct methods
Least-squares matrix: full	Secondary atom site location: difference Fourier map
*R*[*F*^2^ > 2σ(*F*^2^)] = 0.043	Hydrogen site location: inferred from neighbouring sites
*wR*(*F*^2^) = 0.144	H-atom parameters constrained
*S* = 1.07	*w* = 1/[σ^2^(*F*_o_^2^) + (0.0724*P*)^2^ + 0.2771*P*] where *P* = (*F*_o_^2^ + 2*F*_c_^2^)/3
8251 reflections	(Δ/σ)_max_ < 0.001
415 parameters	Δρ_max_ = 0.31 e Å^−^^3^
0 restraints	Δρ_min_ = −0.24 e Å^−^^3^

### Special details

**Table:**

Geometry. All e.s.d.'s (except the e.s.d. in the dihedral angle between two l.s. planes) are estimated using the full covariance matrix. The cell e.s.d.'s are taken into account individually in the estimation of e.s.d.'s in distances, angles and torsion angles; correlations between e.s.d.'s in cell parameters are only used when they are defined by crystal symmetry. An approximate (isotropic) treatment of cell e.s.d.'s is used for estimating e.s.d.'s involving l.s. planes.
Refinement. Refinement of *F*^2^ against ALL reflections. The weighted *R*-factor *wR* and goodness of fit *S* are based on *F*^2^, conventional *R*-factors *R* are based on *F*, with *F* set to zero for negative *F*^2^. The threshold expression of *F*^2^ > σ(*F*^2^) is used only for calculating *R*-factors(gt) *etc*. and is not relevant to the choice of reflections for refinement. *R*-factors based on *F*^2^ are statistically about twice as large as those based on *F*, and *R*- factors based on ALL data will be even larger.

### Fractional atomic coordinates and isotropic or equivalent isotropic displacement parameters (Å^2^)

**Table:**

	*x*	*y*	*z*	*U*_iso_*/*U*_eq_	
S1A	0.67133 (5)	0.82264 (3)	0.08427 (3)	0.04991 (12)	
F1A	0.35358 (17)	0.50191 (10)	0.07601 (10)	0.0780 (4)	
O1A	0.7893 (3)	1.00042 (13)	0.48380 (11)	0.0904 (5)	
N1A	0.57383 (16)	0.74868 (9)	0.27972 (9)	0.0436 (3)	
N2A	0.72337 (16)	0.90516 (9)	0.23250 (9)	0.0419 (3)	
N3A	0.79295 (16)	0.98890 (9)	0.15536 (9)	0.0426 (3)	
C1A	0.51321 (18)	0.68136 (10)	0.23461 (11)	0.0403 (3)	
C2A	0.4170 (2)	0.59282 (12)	0.28661 (13)	0.0490 (3)	
H2AA	0.3889	0.5745	0.3569	0.059*	
C3A	0.3639 (2)	0.53261 (12)	0.23229 (14)	0.0538 (4)	
H3AA	0.2994	0.4733	0.2653	0.065*	
C4A	0.4084 (2)	0.56223 (13)	0.12799 (14)	0.0531 (4)	
C5A	0.5024 (2)	0.64888 (13)	0.07234 (13)	0.0510 (4)	
H5AA	0.5294	0.6664	0.0020	0.061*	
C6A	0.55449 (18)	0.70860 (11)	0.12851 (11)	0.0420 (3)	
C7A	0.65299 (18)	0.82331 (11)	0.20963 (11)	0.0404 (3)	
C8A	0.72749 (19)	0.91363 (12)	0.32581 (11)	0.0446 (3)	
H8AA	0.6862	0.8657	0.3881	0.054*	
C9A	0.80446 (19)	1.00673 (11)	0.31171 (11)	0.0439 (3)	
C10A	0.84341 (18)	1.05089 (11)	0.20331 (11)	0.0406 (3)	
C11A	0.92322 (19)	1.15176 (11)	0.14425 (11)	0.0416 (3)	
C12A	0.8739 (2)	1.20843 (12)	0.04738 (11)	0.0473 (3)	
H12A	0.7925	1.1817	0.0193	0.057*	
C13A	0.9459 (2)	1.30451 (13)	−0.00723 (13)	0.0537 (4)	
H13A	0.9144	1.3414	−0.0725	0.064*	
C14A	1.0642 (2)	1.34576 (13)	0.03492 (14)	0.0561 (4)	
H14A	1.1100	1.4111	−0.0012	0.067*	
C15A	1.1144 (2)	1.29020 (13)	0.13046 (14)	0.0557 (4)	
H15A	1.1949	1.3178	0.1584	0.067*	
C16A	1.0452 (2)	1.19319 (12)	0.18507 (13)	0.0497 (3)	
H16A	1.0804	1.1556	0.2492	0.060*	
C17A	0.8236 (3)	1.04889 (15)	0.39380 (14)	0.0595 (4)	
H17A	0.8642	1.1165	0.3762	0.071*	
S1B	0.85368 (6)	0.49438 (3)	0.24460 (3)	0.05176 (12)	
F1B	0.50654 (18)	0.19249 (10)	0.22056 (12)	0.0855 (4)	
O1B	0.8877 (2)	0.71038 (14)	0.62541 (10)	0.0816 (5)	
N1B	0.71374 (18)	0.43506 (10)	0.43233 (10)	0.0501 (3)	
N2B	0.88353 (17)	0.58379 (10)	0.38940 (10)	0.0473 (3)	
N3B	0.98472 (17)	0.65462 (10)	0.31604 (10)	0.0463 (3)	
C1B	0.65907 (19)	0.36730 (12)	0.38591 (12)	0.0470 (3)	
C2B	0.5465 (2)	0.28536 (14)	0.43263 (15)	0.0589 (4)	
H2BA	0.5057	0.2707	0.5008	0.071*	
C3B	0.4968 (2)	0.22667 (14)	0.37629 (17)	0.0644 (5)	
H3BA	0.4220	0.1718	0.4060	0.077*	
C4B	0.5595 (2)	0.25033 (14)	0.27492 (17)	0.0613 (4)	
C5B	0.6719 (2)	0.32944 (13)	0.22546 (15)	0.0557 (4)	
H5BA	0.7124	0.3430	0.1574	0.067*	
C6B	0.72117 (19)	0.38764 (11)	0.28344 (12)	0.0470 (3)	
C7B	0.8123 (2)	0.50277 (12)	0.36636 (11)	0.0456 (3)	
C8B	0.8587 (2)	0.60645 (13)	0.47682 (12)	0.0510 (4)	
H8BA	0.7943	0.5685	0.5360	0.061*	
C9B	0.9459 (2)	0.69574 (12)	0.46208 (11)	0.0471 (3)	
C10B	1.02340 (19)	0.72353 (12)	0.35926 (11)	0.0435 (3)	
C11B	1.12870 (19)	0.81364 (11)	0.30056 (11)	0.0429 (3)	
C12B	1.1391 (2)	0.84764 (13)	0.19371 (12)	0.0515 (4)	
H12B	1.0808	0.8123	0.1602	0.062*	
C13B	1.2353 (3)	0.93346 (14)	0.13668 (13)	0.0596 (4)	
H13B	1.2417	0.9551	0.0652	0.072*	
C14B	1.3220 (2)	0.98730 (13)	0.18518 (14)	0.0571 (4)	
H14B	1.3867	1.0450	0.1467	0.069*	
C15B	1.3118 (2)	0.95467 (14)	0.29113 (14)	0.0561 (4)	
H15B	1.3683	0.9913	0.3242	0.067*	
C16B	1.2181 (2)	0.86785 (14)	0.34855 (13)	0.0516 (4)	
H16B	1.2148	0.8454	0.4200	0.062*	
C17B	0.9424 (2)	0.74922 (16)	0.53715 (13)	0.0588 (4)	
H17B	0.9844	0.8168	0.5160	0.071*	

### Atomic displacement parameters (Å^2^)

**Table:**

	*U*^11^	*U*^22^	*U*^33^	*U*^12^	*U*^13^	*U*^23^
S1A	0.0640 (3)	0.0439 (2)	0.03977 (19)	−0.01902 (16)	0.00701 (16)	−0.01313 (15)
F1A	0.0888 (9)	0.0728 (7)	0.0857 (8)	−0.0342 (6)	0.0022 (6)	−0.0449 (6)
O1A	0.1431 (16)	0.0870 (11)	0.0442 (7)	−0.0231 (10)	−0.0030 (8)	−0.0259 (7)
N1A	0.0500 (7)	0.0381 (6)	0.0409 (6)	−0.0074 (5)	0.0011 (5)	−0.0118 (5)
N2A	0.0480 (7)	0.0372 (6)	0.0393 (6)	−0.0097 (5)	0.0003 (5)	−0.0117 (5)
N3A	0.0519 (7)	0.0356 (6)	0.0389 (6)	−0.0107 (5)	0.0024 (5)	−0.0115 (5)
C1A	0.0415 (7)	0.0334 (6)	0.0437 (7)	−0.0043 (5)	0.0005 (5)	−0.0106 (5)
C2A	0.0535 (9)	0.0396 (7)	0.0485 (8)	−0.0103 (6)	0.0061 (6)	−0.0098 (6)
C3A	0.0513 (9)	0.0413 (8)	0.0653 (10)	−0.0146 (6)	0.0036 (7)	−0.0143 (7)
C4A	0.0516 (9)	0.0478 (8)	0.0667 (10)	−0.0104 (7)	−0.0039 (7)	−0.0279 (8)
C5A	0.0564 (9)	0.0506 (8)	0.0479 (8)	−0.0113 (7)	0.0011 (7)	−0.0199 (7)
C6A	0.0449 (7)	0.0376 (7)	0.0423 (7)	−0.0069 (5)	0.0013 (5)	−0.0127 (5)
C7A	0.0441 (7)	0.0365 (6)	0.0403 (7)	−0.0054 (5)	−0.0003 (5)	−0.0130 (5)
C8A	0.0508 (8)	0.0418 (7)	0.0390 (7)	−0.0071 (6)	−0.0019 (6)	−0.0106 (6)
C9A	0.0511 (8)	0.0423 (7)	0.0395 (7)	−0.0059 (6)	−0.0036 (6)	−0.0148 (6)
C10A	0.0447 (7)	0.0360 (6)	0.0414 (7)	−0.0047 (5)	−0.0011 (5)	−0.0139 (5)
C11A	0.0476 (8)	0.0350 (6)	0.0433 (7)	−0.0061 (5)	0.0027 (5)	−0.0158 (5)
C12A	0.0548 (9)	0.0464 (8)	0.0422 (7)	−0.0091 (6)	−0.0001 (6)	−0.0174 (6)
C13A	0.0658 (10)	0.0469 (8)	0.0436 (8)	−0.0075 (7)	0.0023 (7)	−0.0100 (6)
C14A	0.0666 (10)	0.0429 (8)	0.0555 (9)	−0.0155 (7)	0.0095 (7)	−0.0150 (7)
C15A	0.0569 (10)	0.0516 (9)	0.0602 (10)	−0.0178 (7)	0.0006 (7)	−0.0211 (8)
C16A	0.0547 (9)	0.0441 (8)	0.0501 (8)	−0.0100 (6)	−0.0060 (7)	−0.0147 (6)
C17A	0.0771 (12)	0.0576 (10)	0.0486 (9)	−0.0107 (8)	−0.0045 (8)	−0.0238 (8)
S1B	0.0601 (2)	0.0447 (2)	0.0457 (2)	−0.01154 (16)	0.01026 (16)	−0.01161 (16)
F1B	0.0844 (9)	0.0729 (8)	0.1136 (11)	−0.0191 (6)	−0.0036 (7)	−0.0501 (7)
O1B	0.0912 (11)	0.1110 (12)	0.0449 (7)	−0.0069 (9)	0.0001 (7)	−0.0300 (7)
N1B	0.0538 (8)	0.0477 (7)	0.0411 (7)	−0.0078 (6)	0.0009 (5)	−0.0055 (5)
N2B	0.0536 (7)	0.0438 (7)	0.0395 (6)	−0.0079 (5)	0.0017 (5)	−0.0082 (5)
N3B	0.0508 (7)	0.0423 (6)	0.0416 (6)	−0.0077 (5)	0.0038 (5)	−0.0100 (5)
C1B	0.0436 (8)	0.0418 (7)	0.0485 (8)	−0.0033 (6)	−0.0005 (6)	−0.0064 (6)
C2B	0.0522 (9)	0.0516 (9)	0.0618 (10)	−0.0110 (7)	0.0068 (7)	−0.0064 (8)
C3B	0.0509 (10)	0.0503 (9)	0.0847 (13)	−0.0122 (7)	0.0045 (8)	−0.0147 (9)
C4B	0.0535 (10)	0.0500 (9)	0.0849 (13)	−0.0030 (7)	−0.0055 (9)	−0.0284 (9)
C5B	0.0569 (10)	0.0497 (9)	0.0614 (10)	−0.0032 (7)	0.0031 (7)	−0.0217 (8)
C6B	0.0461 (8)	0.0388 (7)	0.0506 (8)	−0.0020 (6)	0.0022 (6)	−0.0093 (6)
C7B	0.0476 (8)	0.0416 (7)	0.0416 (7)	−0.0036 (6)	−0.0012 (6)	−0.0064 (6)
C8B	0.0564 (9)	0.0551 (9)	0.0362 (7)	−0.0053 (7)	−0.0006 (6)	−0.0087 (6)
C9B	0.0497 (8)	0.0508 (8)	0.0371 (7)	−0.0019 (6)	−0.0037 (6)	−0.0097 (6)
C10B	0.0444 (7)	0.0441 (7)	0.0395 (7)	−0.0004 (6)	−0.0023 (5)	−0.0110 (6)
C11B	0.0448 (7)	0.0405 (7)	0.0432 (7)	−0.0016 (5)	−0.0007 (5)	−0.0141 (6)
C12B	0.0639 (10)	0.0483 (8)	0.0436 (8)	−0.0104 (7)	0.0013 (7)	−0.0182 (6)
C13B	0.0790 (12)	0.0520 (9)	0.0446 (9)	−0.0138 (8)	0.0043 (8)	−0.0134 (7)
C14B	0.0620 (10)	0.0462 (8)	0.0606 (10)	−0.0116 (7)	0.0028 (8)	−0.0157 (7)
C15B	0.0537 (9)	0.0565 (9)	0.0639 (10)	−0.0101 (7)	−0.0042 (7)	−0.0274 (8)
C16B	0.0532 (9)	0.0570 (9)	0.0458 (8)	−0.0044 (7)	−0.0055 (6)	−0.0182 (7)
C17B	0.0604 (10)	0.0719 (11)	0.0472 (9)	−0.0051 (8)	−0.0039 (7)	−0.0241 (8)

### Geometric parameters (Å, °)

**Table:**

S1A—C7A	1.7331 (15)	S1B—C6B	1.7307 (16)
S1A—C6A	1.7344 (14)	S1B—C7B	1.7330 (16)
F1A—C4A	1.3626 (18)	F1B—C4B	1.356 (2)
O1A—C17A	1.210 (2)	O1B—C17B	1.206 (2)
N1A—C7A	1.2831 (17)	N1B—C7B	1.2896 (19)
N1A—C1A	1.3882 (18)	N1B—C1B	1.387 (2)
N2A—C8A	1.3385 (19)	N2B—C8B	1.346 (2)
N2A—N3A	1.3667 (15)	N2B—N3B	1.3668 (16)
N2A—C7A	1.4010 (18)	N2B—C7B	1.395 (2)
N3A—C10A	1.3258 (18)	N3B—C10B	1.327 (2)
C1A—C2A	1.3934 (19)	C1B—C2B	1.397 (2)
C1A—C6A	1.402 (2)	C1B—C6B	1.402 (2)
C2A—C3A	1.381 (2)	C2B—C3B	1.377 (3)
C2A—H2AA	0.9300	C2B—H2BA	0.9300
C3A—C4A	1.381 (2)	C3B—C4B	1.384 (3)
C3A—H3AA	0.9300	C3B—H3BA	0.9300
C4A—C5A	1.376 (2)	C4B—C5B	1.378 (2)
C5A—C6A	1.393 (2)	C5B—C6B	1.388 (2)
C5A—H5AA	0.9300	C5B—H5BA	0.9300
C8A—C9A	1.372 (2)	C8B—C9B	1.366 (2)
C8A—H8AA	0.9300	C8B—H8BA	0.9300
C9A—C10A	1.427 (2)	C9B—C10B	1.436 (2)
C9A—C17A	1.456 (2)	C9B—C17B	1.456 (2)
C10A—C11A	1.4730 (19)	C10B—C11B	1.467 (2)
C11A—C12A	1.393 (2)	C11B—C12B	1.390 (2)
C11A—C16A	1.393 (2)	C11B—C16B	1.393 (2)
C12A—C13A	1.385 (2)	C12B—C13B	1.382 (2)
C12A—H12A	0.9300	C12B—H12B	0.9300
C13A—C14A	1.381 (3)	C13B—C14B	1.383 (2)
C13A—H13A	0.9300	C13B—H13B	0.9300
C14A—C15A	1.377 (3)	C14B—C15B	1.378 (3)
C14A—H14A	0.9300	C14B—H14B	0.9300
C15A—C16A	1.387 (2)	C15B—C16B	1.382 (2)
C15A—H15A	0.9300	C15B—H15B	0.9300
C16A—H16A	0.9300	C16B—H16B	0.9300
C17A—H17A	0.9300	C17B—H17B	0.9300
			
C7A—S1A—C6A	87.24 (7)	C6B—S1B—C7B	87.38 (7)
C7A—N1A—C1A	108.70 (12)	C7B—N1B—C1B	108.42 (13)
C8A—N2A—N3A	113.04 (12)	C8B—N2B—N3B	112.64 (13)
C8A—N2A—C7A	126.58 (12)	C8B—N2B—C7B	127.64 (13)
N3A—N2A—C7A	120.35 (11)	N3B—N2B—C7B	119.62 (13)
C10A—N3A—N2A	104.40 (11)	C10B—N3B—N2B	104.84 (12)
N1A—C1A—C2A	124.95 (13)	N1B—C1B—C2B	124.95 (15)
N1A—C1A—C6A	114.96 (12)	N1B—C1B—C6B	115.21 (13)
C2A—C1A—C6A	120.08 (14)	C2B—C1B—C6B	119.80 (16)
C3A—C2A—C1A	119.11 (15)	C3B—C2B—C1B	119.08 (17)
C3A—C2A—H2AA	120.4	C3B—C2B—H2BA	120.5
C1A—C2A—H2AA	120.4	C1B—C2B—H2BA	120.5
C2A—C3A—C4A	118.72 (14)	C2B—C3B—C4B	119.22 (16)
C2A—C3A—H3AA	120.6	C2B—C3B—H3BA	120.4
C4A—C3A—H3AA	120.6	C4B—C3B—H3BA	120.4
F1A—C4A—C5A	117.53 (16)	F1B—C4B—C5B	117.80 (18)
F1A—C4A—C3A	117.59 (14)	F1B—C4B—C3B	118.10 (17)
C5A—C4A—C3A	124.87 (15)	C5B—C4B—C3B	124.10 (18)
C4A—C5A—C6A	115.49 (15)	C4B—C5B—C6B	115.96 (17)
C4A—C5A—H5AA	122.3	C4B—C5B—H5BA	122.0
C6A—C5A—H5AA	122.3	C6B—C5B—H5BA	122.0
C5A—C6A—C1A	121.72 (13)	C5B—C6B—C1B	121.84 (15)
C5A—C6A—S1A	128.22 (12)	C5B—C6B—S1B	128.06 (13)
C1A—C6A—S1A	110.06 (10)	C1B—C6B—S1B	110.06 (12)
N1A—C7A—N2A	121.30 (13)	N1B—C7B—N2B	121.97 (15)
N1A—C7A—S1A	119.01 (11)	N1B—C7B—S1B	118.92 (13)
N2A—C7A—S1A	119.69 (10)	N2B—C7B—S1B	119.10 (11)
N2A—C8A—C9A	106.68 (13)	N2B—C8B—C9B	106.95 (14)
N2A—C8A—H8AA	126.7	N2B—C8B—H8BA	126.5
C9A—C8A—H8AA	126.7	C9B—C8B—H8BA	126.5
C8A—C9A—C10A	105.00 (13)	C8B—C9B—C10B	105.11 (14)
C8A—C9A—C17A	124.08 (14)	C8B—C9B—C17B	123.68 (15)
C10A—C9A—C17A	130.72 (14)	C10B—C9B—C17B	131.06 (15)
N3A—C10A—C9A	110.88 (12)	N3B—C10B—C9B	110.46 (13)
N3A—C10A—C11A	120.38 (12)	N3B—C10B—C11B	119.89 (13)
C9A—C10A—C11A	128.72 (13)	C9B—C10B—C11B	129.63 (14)
C12A—C11A—C16A	119.04 (13)	C12B—C11B—C16B	118.35 (14)
C12A—C11A—C10A	120.05 (13)	C12B—C11B—C10B	119.60 (14)
C16A—C11A—C10A	120.88 (14)	C16B—C11B—C10B	122.04 (14)
C13A—C12A—C11A	120.16 (15)	C13B—C12B—C11B	120.69 (15)
C13A—C12A—H12A	119.9	C13B—C12B—H12B	119.7
C11A—C12A—H12A	119.9	C11B—C12B—H12B	119.7
C14A—C13A—C12A	120.28 (16)	C12B—C13B—C14B	120.43 (16)
C14A—C13A—H13A	119.9	C12B—C13B—H13B	119.8
C12A—C13A—H13A	119.9	C14B—C13B—H13B	119.8
C15A—C14A—C13A	120.04 (15)	C15B—C14B—C13B	119.39 (16)
C15A—C14A—H14A	120.0	C15B—C14B—H14B	120.3
C13A—C14A—H14A	120.0	C13B—C14B—H14B	120.3
C14A—C15A—C16A	120.16 (16)	C14B—C15B—C16B	120.42 (16)
C14A—C15A—H15A	119.9	C14B—C15B—H15B	119.8
C16A—C15A—H15A	119.9	C16B—C15B—H15B	119.8
C15A—C16A—C11A	120.30 (15)	C15B—C16B—C11B	120.69 (15)
C15A—C16A—H16A	119.9	C15B—C16B—H16B	119.7
C11A—C16A—H16A	119.9	C11B—C16B—H16B	119.7
O1A—C17A—C9A	123.28 (17)	O1B—C17B—C9B	123.33 (19)
O1A—C17A—H17A	118.4	O1B—C17B—H17B	118.3
C9A—C17A—H17A	118.4	C9B—C17B—H17B	118.3
			
C8A—N2A—N3A—C10A	0.64 (17)	C8B—N2B—N3B—C10B	−0.18 (18)
C7A—N2A—N3A—C10A	178.86 (13)	C7B—N2B—N3B—C10B	176.45 (14)
C7A—N1A—C1A—C2A	−178.13 (15)	C7B—N1B—C1B—C2B	−177.98 (16)
C7A—N1A—C1A—C6A	1.36 (19)	C7B—N1B—C1B—C6B	0.1 (2)
N1A—C1A—C2A—C3A	179.70 (15)	N1B—C1B—C2B—C3B	177.10 (16)
C6A—C1A—C2A—C3A	0.2 (2)	C6B—C1B—C2B—C3B	−0.9 (3)
C1A—C2A—C3A—C4A	0.1 (3)	C1B—C2B—C3B—C4B	0.0 (3)
C2A—C3A—C4A—F1A	−179.57 (16)	C2B—C3B—C4B—F1B	−178.94 (17)
C2A—C3A—C4A—C5A	−0.4 (3)	C2B—C3B—C4B—C5B	0.8 (3)
F1A—C4A—C5A—C6A	179.47 (15)	F1B—C4B—C5B—C6B	179.16 (16)
C3A—C4A—C5A—C6A	0.3 (3)	C3B—C4B—C5B—C6B	−0.5 (3)
C4A—C5A—C6A—C1A	0.1 (2)	C4B—C5B—C6B—C1B	−0.4 (3)
C4A—C5A—C6A—S1A	−179.15 (13)	C4B—C5B—C6B—S1B	−177.55 (14)
N1A—C1A—C6A—C5A	−179.85 (15)	N1B—C1B—C6B—C5B	−177.06 (15)
C2A—C1A—C6A—C5A	−0.3 (2)	C2B—C1B—C6B—C5B	1.1 (2)
N1A—C1A—C6A—S1A	−0.51 (17)	N1B—C1B—C6B—S1B	0.56 (18)
C2A—C1A—C6A—S1A	179.01 (12)	C2B—C1B—C6B—S1B	178.70 (13)
C7A—S1A—C6A—C5A	178.96 (16)	C7B—S1B—C6B—C5B	176.70 (17)
C7A—S1A—C6A—C1A	−0.33 (11)	C7B—S1B—C6B—C1B	−0.73 (12)
C1A—N1A—C7A—N2A	177.85 (13)	C1B—N1B—C7B—N2B	177.88 (14)
C1A—N1A—C7A—S1A	−1.70 (17)	C1B—N1B—C7B—S1B	−0.70 (19)
C8A—N2A—C7A—N1A	4.4 (2)	C8B—N2B—C7B—N1B	−2.7 (3)
N3A—N2A—C7A—N1A	−173.56 (13)	N3B—N2B—C7B—N1B	−178.78 (15)
C8A—N2A—C7A—S1A	−176.06 (12)	C8B—N2B—C7B—S1B	175.86 (13)
N3A—N2A—C7A—S1A	5.98 (19)	N3B—N2B—C7B—S1B	−0.2 (2)
C6A—S1A—C7A—N1A	1.24 (13)	C6B—S1B—C7B—N1B	0.88 (14)
C6A—S1A—C7A—N2A	−178.32 (13)	C6B—S1B—C7B—N2B	−177.74 (14)
N3A—N2A—C8A—C9A	−0.53 (18)	N3B—N2B—C8B—C9B	0.05 (19)
C7A—N2A—C8A—C9A	−178.62 (15)	C7B—N2B—C8B—C9B	−176.25 (15)
N2A—C8A—C9A—C10A	0.20 (17)	N2B—C8B—C9B—C10B	0.10 (18)
N2A—C8A—C9A—C17A	175.54 (16)	N2B—C8B—C9B—C17B	176.03 (16)
N2A—N3A—C10A—C9A	−0.49 (17)	N2B—N3B—C10B—C9B	0.24 (17)
N2A—N3A—C10A—C11A	−178.81 (13)	N2B—N3B—C10B—C11B	−178.39 (13)
C8A—C9A—C10A—N3A	0.19 (18)	C8B—C9B—C10B—N3B	−0.22 (19)
C17A—C9A—C10A—N3A	−174.72 (17)	C17B—C9B—C10B—N3B	−175.73 (17)
C8A—C9A—C10A—C11A	178.33 (15)	C8B—C9B—C10B—C11B	178.24 (16)
C17A—C9A—C10A—C11A	3.4 (3)	C17B—C9B—C10B—C11B	2.7 (3)
N3A—C10A—C11A—C12A	34.0 (2)	N3B—C10B—C11B—C12B	22.9 (2)
C9A—C10A—C11A—C12A	−143.94 (16)	C9B—C10B—C11B—C12B	−155.43 (17)
N3A—C10A—C11A—C16A	−147.70 (15)	N3B—C10B—C11B—C16B	−157.91 (16)
C9A—C10A—C11A—C16A	34.3 (2)	C9B—C10B—C11B—C16B	23.8 (3)
C16A—C11A—C12A—C13A	0.1 (2)	C16B—C11B—C12B—C13B	−0.3 (3)
C10A—C11A—C12A—C13A	178.39 (14)	C10B—C11B—C12B—C13B	178.89 (16)
C11A—C12A—C13A—C14A	−1.5 (3)	C11B—C12B—C13B—C14B	−0.4 (3)
C12A—C13A—C14A—C15A	1.7 (3)	C12B—C13B—C14B—C15B	0.0 (3)
C13A—C14A—C15A—C16A	−0.7 (3)	C13B—C14B—C15B—C16B	1.1 (3)
C14A—C15A—C16A—C11A	−0.7 (3)	C14B—C15B—C16B—C11B	−1.9 (3)
C12A—C11A—C16A—C15A	1.0 (2)	C12B—C11B—C16B—C15B	1.4 (3)
C10A—C11A—C16A—C15A	−177.30 (15)	C10B—C11B—C16B—C15B	−177.75 (15)
C8A—C9A—C17A—O1A	8.3 (3)	C8B—C9B—C17B—O1B	14.1 (3)
C10A—C9A—C17A—O1A	−177.6 (2)	C10B—C9B—C17B—O1B	−171.10 (19)

### Hydrogen-bond geometry (Å, °)

**Table:**

*Cg*4 is the centroid of the C11B–C16B ring.

**Table:**

*D*—H···*A*	*D*—H	H···*A*	*D*···*A*	*D*—H···*A*
C5B—H5BA···Cg4^i^	0.93	2.85	3.4757 (19)	126

Symmetry codes: (i) *x*, *y*−1, *z*.
